# Supplementary material for: Differential COVID-19 Outcomes Across Lysosomal Disorders
Source: medRxiv. 2026 Jun 24:2026.06.22.26356274. Preprint. [Version 1] doi: 10.64898/2026.06.22.26356274 (PMC13321181; doi:10.64898/2026.06.22.26356274)
Supplement: Supplementary file 1 [file media-1.pdf]

Differential COVID-19 Outcomes Across Lysosomal Disorders

Supplementary Material

Blake K. Byer[1], Zachary Butzin-Dozier[2], Brenda M. McGrath[3], Joseph Muenzer[4], Lorne Clarke[5], Melissa A. Haendel[1], Shawn T. O'Neil[1*]

[1]Department of Genetics, University of North Carolina at Chapel Hill, Chapel Hill, North Carolina, United States
[2]Department of Pediatrics, Stanford University School of Medicine, Stanford, California, United States
[3]OCHIN, Portland, Oregon, United States
[4]Department of Pediatrics, University of North Carolina at Chapel Hill, Chapel Hill, North Carolina, United States
[5]Department of Medical Genetics, University of British Columbia, Vancouver, British Columbia, Canada
[*]Corresponding author (oneilsh@unc.edu)

Supplementary Figures

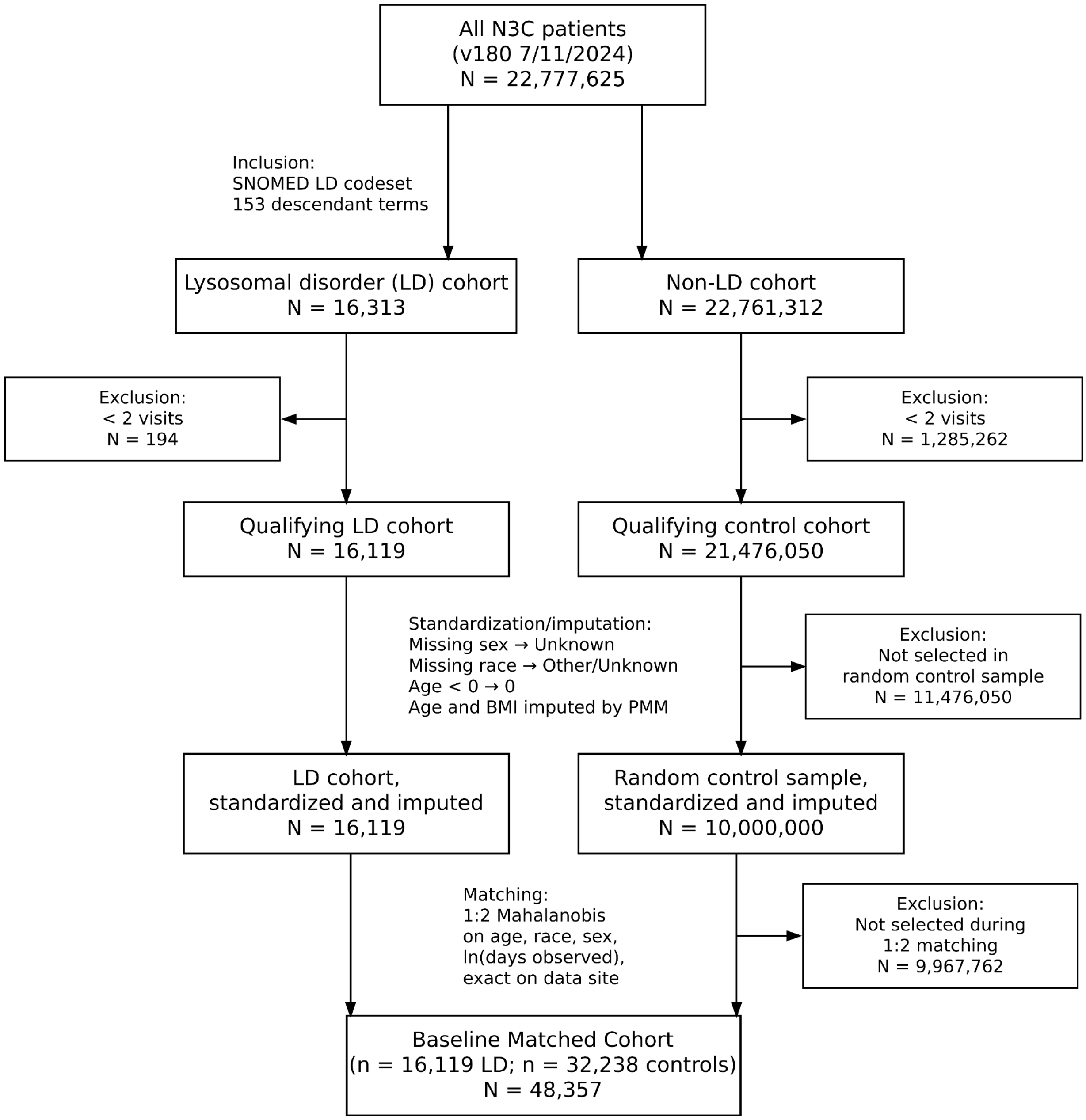

Supplementary Figure 1: Cohort construction of the Baseline Matched Cohort. Flowchart of inclusion and exclusion criteria applied to the N3C population. Before imputation, in LDs, age was 4.9% missing, and BMI was 11% missing; in controls, age was 4.8% missing, and BMI was 27% missing. Standardization, imputation, and 1:2 Mahalanobis matching were performed to construct a cohort comprising 16,119 patients with LDs and 32,238 controls.

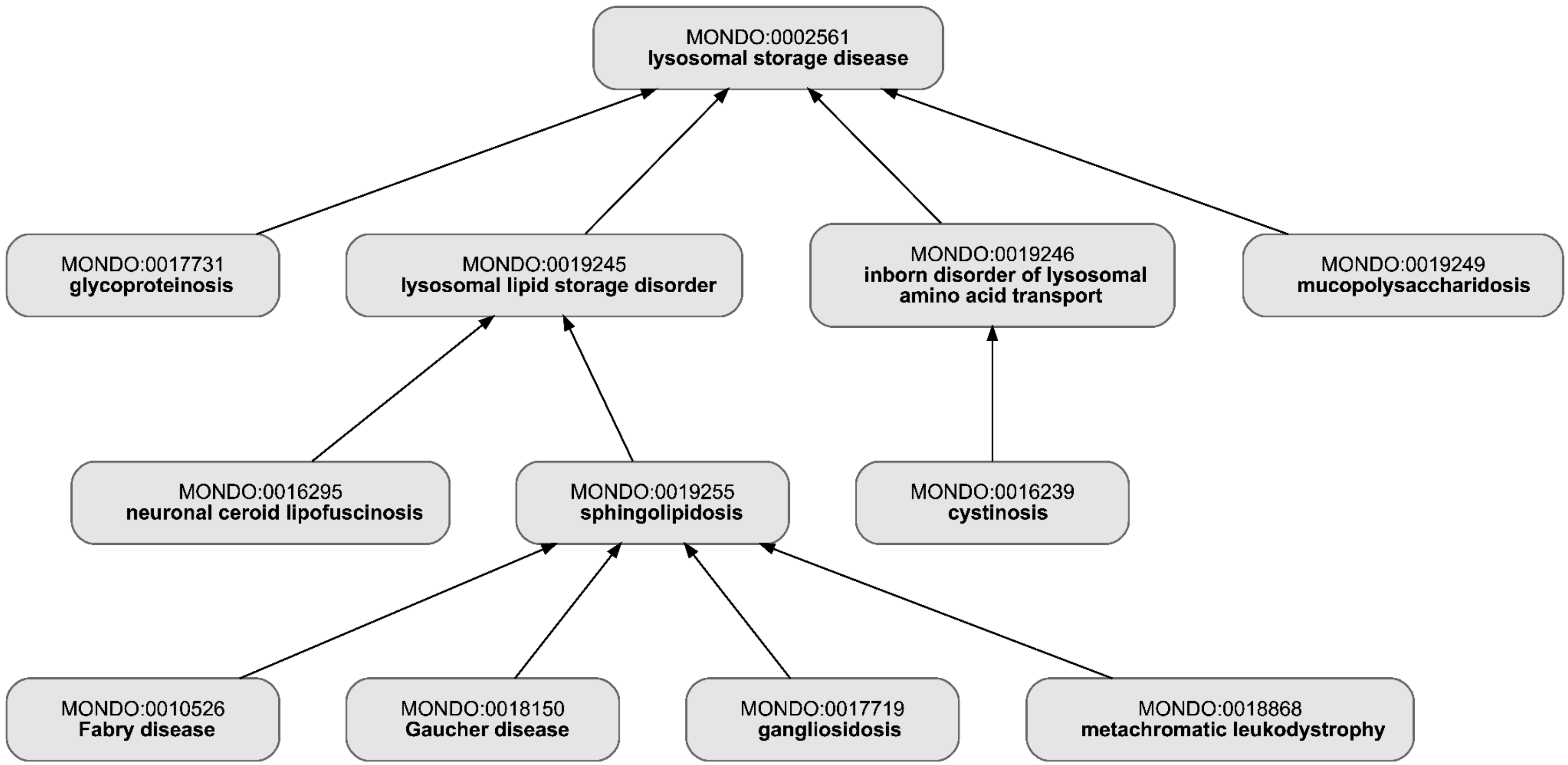

Supplementary Figure 2: Mondo Disease Ontology lysosomal disorders hierarchy.
Nodes represent Mondo terms (ID and label), and edges denote parent–child relationships. This hierarchy was used to define non-mutually exclusive category groupings for regression analyses.

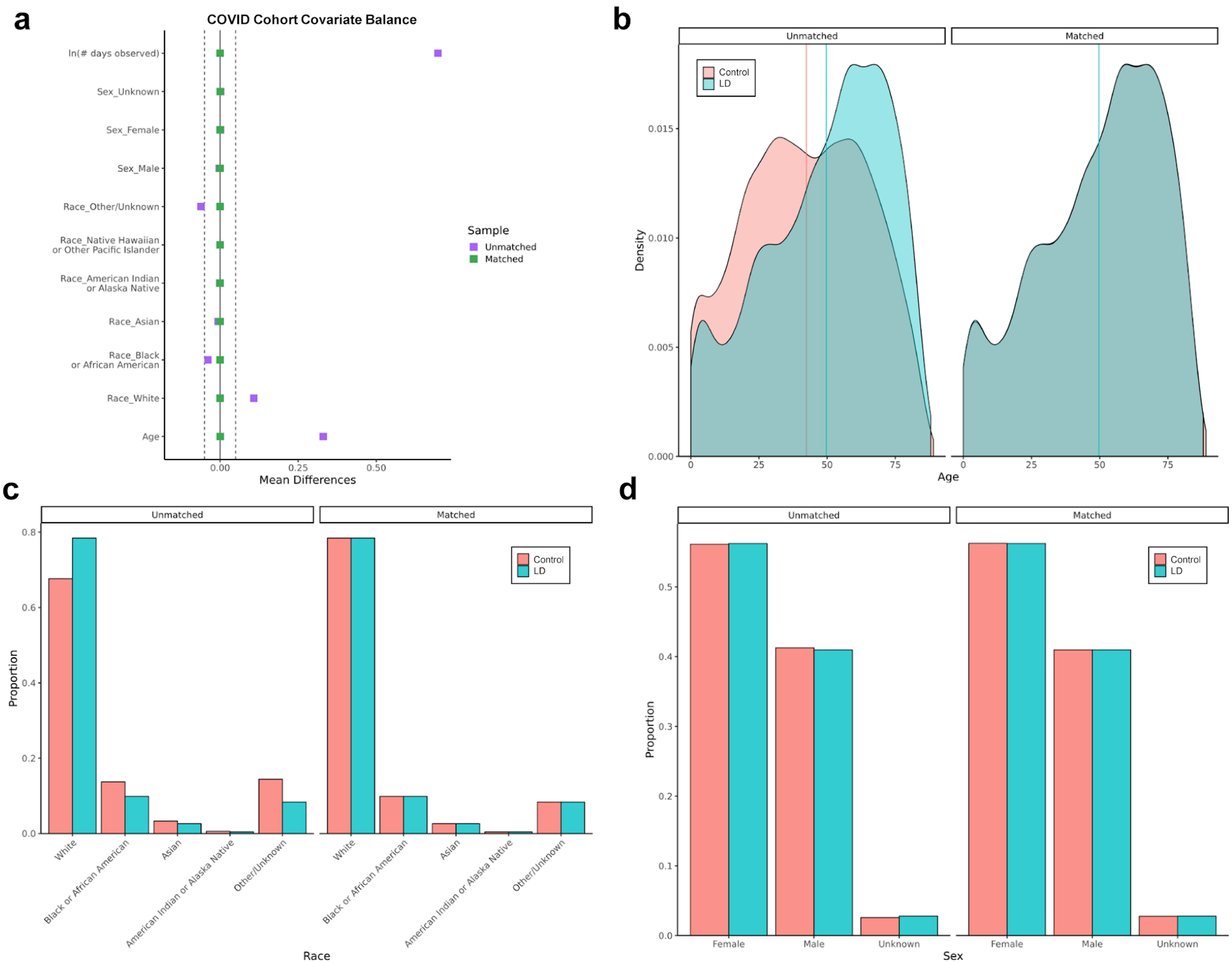
a
COVID Cohort Covariate Balance
ln(# days observed)
Sex_Unknown
Sex_Female
Sex_Male
Race_Other/Unknown
Race_Native Hawaiian or Other Pacific Islander
Race_American Indian or Alaska Native
Race_Asian
Race_Black or African American
Race_White
Age
0.00
0.25
0.50
Mean Differences
Sample
Unmatched
Matched
b
Unmatched
Matched
Control
LD
Density
0.000
0.005
0.010
0.015
0
25
50
75
Age
c
Unmatched
Matched
Control
LD
Proportion
0.0
0.2
0.4
0.6
0.8
White
Black or African American
Asian
American Indian or Alaska Native
Other/Unknown
Race
d
Unmatched
Matched
Control
LD
Proportion
0.0
0.1
0.2
0.3
0.4
0.5
Female
Male
Unknown
Sex

Supplementary Figure 3: COVID Matched Cohort covariate balance before and after Mahalanobis distance matching
a, Love plot displaying standardized mean differences (SMD) for demographic variables (age, race, sex) and a clinical utilization measure (ln[# days observed pre-COVID]) for unmatched (purple) and Mahalanobis-distance matched (green) samples. Vertical dashed lines mark the ±0.1 SMD threshold for negligible imbalance.
b, Age density distributions for LD (teal) and control (coral) groups, showing the reduction in age-related bias following Mahalanobis distance matching. Vertical lines indicate median age.
c, d, Proportional distribution of race (**c**) and sex (**d**), demonstrating the effectiveness of Mahalanobis distance matching at neutralizing demographic disparities and accounting for covariate covariance.

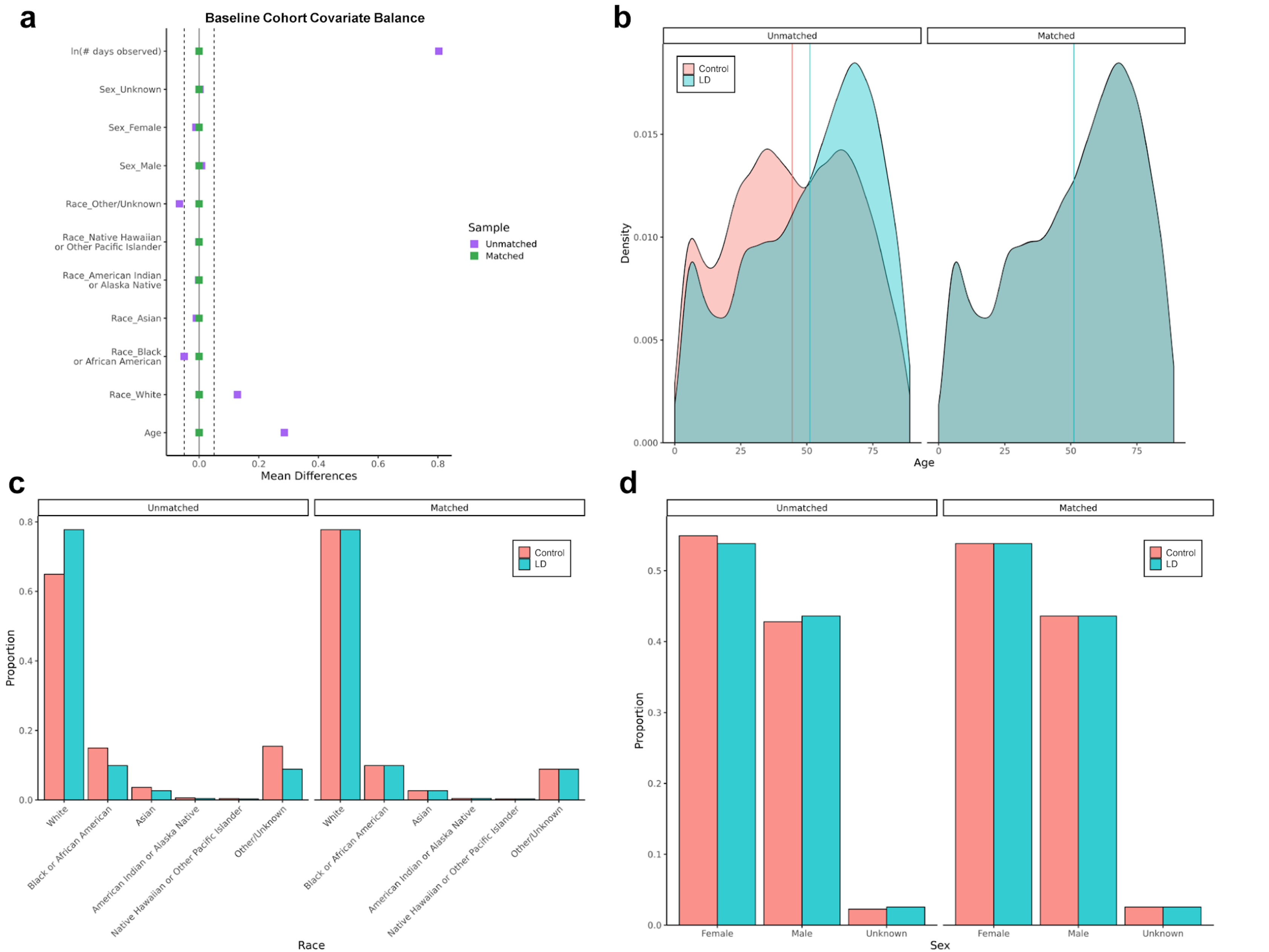

a
Baseline Cohort Covariate Balance
ln(# days observed)
Sex_Unknown
Sex_Female
Sex_Male
Race_Other/Unknown
Race_Native Hawaiian or Other Pacific Islander
Race_American Indian or Alaska Native
Race_Asian
Race_Black or African American
Race_White
Age
0.0
0.2
0.4
0.6
0.8
Mean Differences
Sample
Unmatched
Matched
b
Unmatched
Matched
Control
LD
Density
0.000
0.005
0.010
0.015
0
25
50
75
Age
c
Unmatched
Matched
Control
LD
Proportion
0.0
0.2
0.4
0.6
0.8
White
Black or African American
Asian
American Indian or Alaska Native
Native Hawaiian or Other Pacific Islander
Other/Unknown
Race
d
Unmatched
Matched
Control
LD
Proportion
0.0
0.1
0.2
0.3
0.4
0.5
Female
Male
Unknown
Sex

Supplementary Figure 4: Baseline Matched Cohort covariate balance before and after Mahalanobis distance matching.
a-d, Standardized mean differences and demographic distributions for the Baseline Matched Cohort. Presentation and color schemes follow the conventions described in Supplementary Figure 3. Matching effectively neutralized disparities in age, race, and sex within the baseline population.

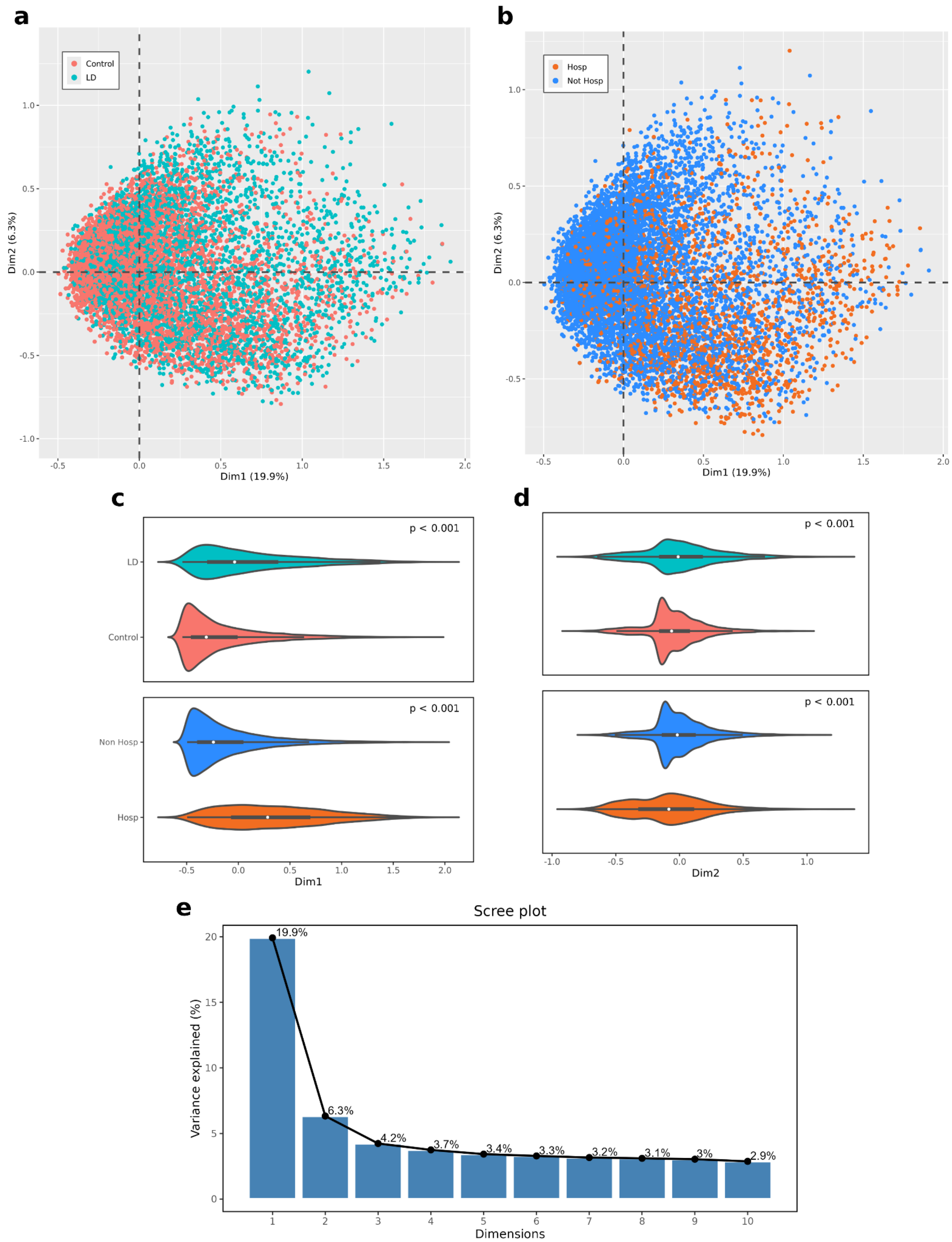

Supplementary Figure 5: Multiple correspondence analysis of lysosomal disorder status and COVID-19 hospitalization.

a, MCA score plot of the first two principal components (Dim1 and Dim2) colored by disease status (LD, teal; control, coral). **b,** Score plot colored by COVID-19 severity (hospitalized, orange; non-hospitalized, blue).
c, d, Marginal distributions of individuals along Dimension 1 (Dim1) (**c**) and Dimension 2 (Dim2) (**d**), stratified by lysosomal disorder status (top) and hospitalization (bottom). Violin plots illustrate the distribution of patients with median score and IQR indicated by embedded boxplots; differences between groups were assessed using a two-sided Wilcoxon rank-sum test, with LD and hospitalized patients showing significant shifts along both dimensions ($p < 0.001$).
e, Scree plot displaying the percentage of variance explained by the first 10 dimensions. The first dimension (Dim1) accounts for 19.9% of the total variance, while the second (Dim2) accounts for 6.3%, with a noticeable drop-off in variance captured thereafter.

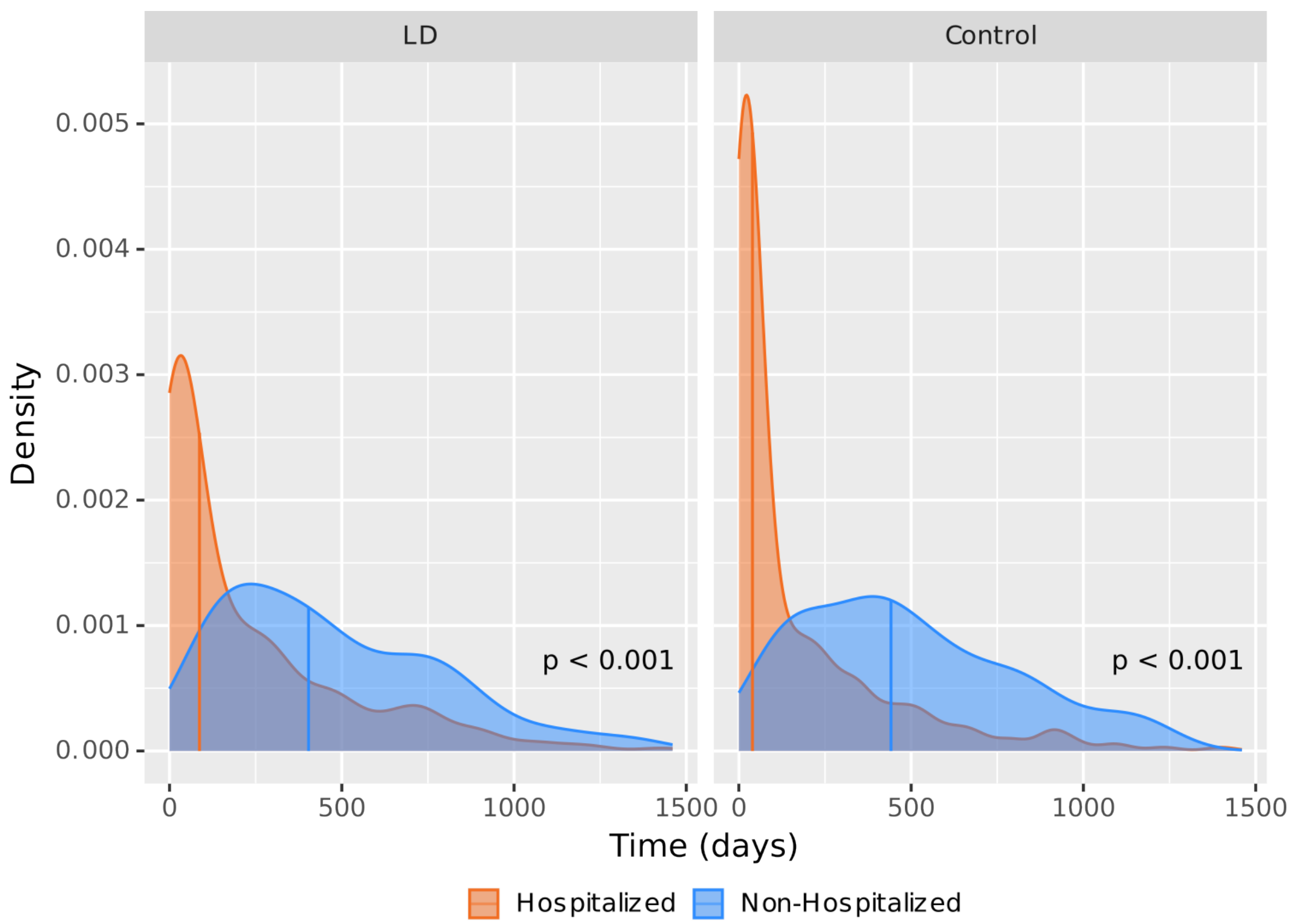

Supplementary Figure 6: Density of Time-to-Death Post-COVID.
Probability density distribution of time-to-death for hospitalized (orange) vs non-hospitalized (blue) across LD (left) and control (right) groups. Vertical lines indicate the median time-to-death for each group (hospitalized LD/control: 87 vs 40 days; non-hospitalized LD/control: 404 vs 442 days). P-values determined by a two-sided Wilcoxon rank-sum test comparing hospitalized and non-hospitalized patients in each panel.

Subgroup	N	HR (95% CI)	p-value
**Albuterol**			
Drug (Control)	821	3.22 (2.08, 4.98)	< 0.001
Drug × LD	653	0.60 (0.32, 1.13)	0.113
**Apixaban**			
Drug (Control)	204	6.22 (2.73, 14.18)	< 0.001
Drug × LD	173	0.34 (0.11, 1.01)	0.052
**Aspirin**			
Drug (Control)	428	5.47 (3.24, 9.24)	< 0.001
Drug × LD	352	0.32 (0.15, 0.69)	0.004
**Budesonide**			
Drug (Control)	140	6.00 (2.24, 16.04)	< 0.001
Drug × LD	156	0.31 (0.08, 1.24)	0.098
**Carvedilol**			
Drug (Control)	110	2.86 (0.92, 8.89)	0.069
Drug × LD	112	1.38 (0.24, 8.07)	0.720
**Dexamethasone**			
Drug (Control)	525	6.77 (3.99, 11.50)	< 0.001
Drug × LD	423	0.43 (0.20, 0.91)	0.027
**Famotidine**			
Drug (Control)	382	3.17 (1.72, 5.84)	< 0.001
Drug × LD	284	0.66 (0.25, 1.69)	0.383
**Heparin**			
Drug (Control)	271	6.58 (3.28, 13.20)	< 0.001
Drug × LD	257	0.57 (0.22, 1.48)	0.249
**Insulin**			
Drug (Control)	430	6.27 (3.63, 10.81)	< 0.001
Drug × LD	404	0.74 (0.34, 1.59)	0.441
**Lisinopril**			
Drug (Control)	233	4.89 (2.10, 11.38)	< 0.001
Drug × LD	146	0.47 (0.12, 1.92)	0.294
**Methylprednisolone**			
Drug (Control)	129	3.08 (1.06, 8.96)	0.039
Drug × LD	118	0.67 (0.16, 2.82)	0.586
**Nirmatrelvir**			
Drug (Control)	189	0.48 (0.10, 2.29)	0.360
Drug × LD	91	2.24 (0.17, 28.66)	0.535
**Prednisone**			
Drug (Control)	275	1.36 (0.63, 2.92)	0.432
Drug × LD	216	2.97 (0.89, 9.95)	0.077
**Remdesivir**			
Drug (Control)	357	5.01 (2.82, 8.93)	< 0.001
Drug × LD	297	0.42 (0.19, 0.95)	0.037
**Systemic Hydrocortisone**			
Drug (Control)	176	2.59 (0.80, 8.39)	0.112
Drug × LD	130	0.34 (0.08, 1.48)	0.151

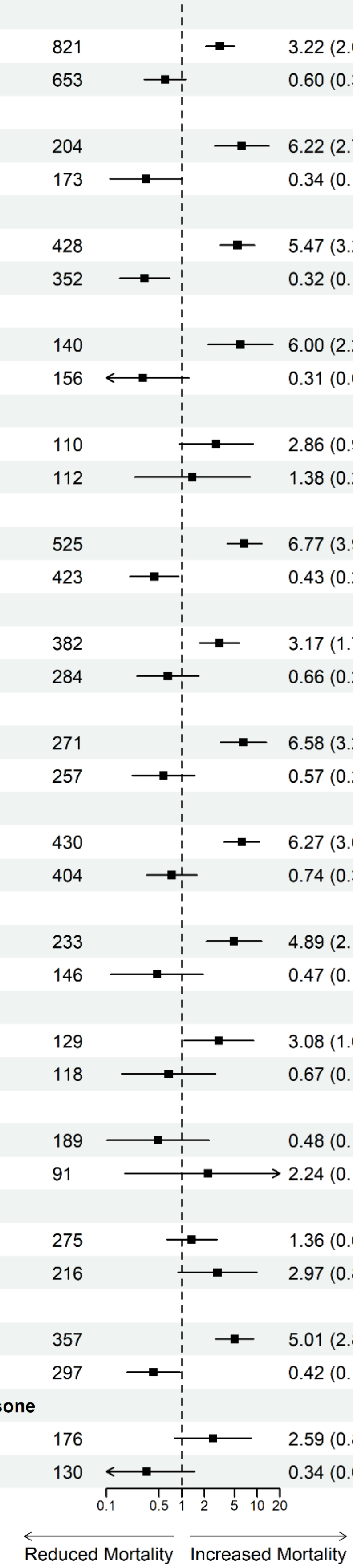

Supplementary Figure 7: Association between acute-phase drugs and mortality with LD interaction effects.

Forest plot showing hazard ratios (HR) and 95% confidence intervals from Cox proportional hazards models evaluating the association between administration of selected drugs and mortality. Estimates are shown for control patients (“Drug (Control)”) and for the interaction term between drug exposure and LD status (“Drug × LD”). Models were adjusted for Elixhauser comorbidity score and stratified by matched subclass. The vertical dashed line indicates HR = 1. Sample sizes (N) are displayed; for “Drug × LD,” N reflects the number of patients with LDs receiving the drug and does not necessarily correspond to the effective sample size used in model estimation. Corresponding p-values are shown.

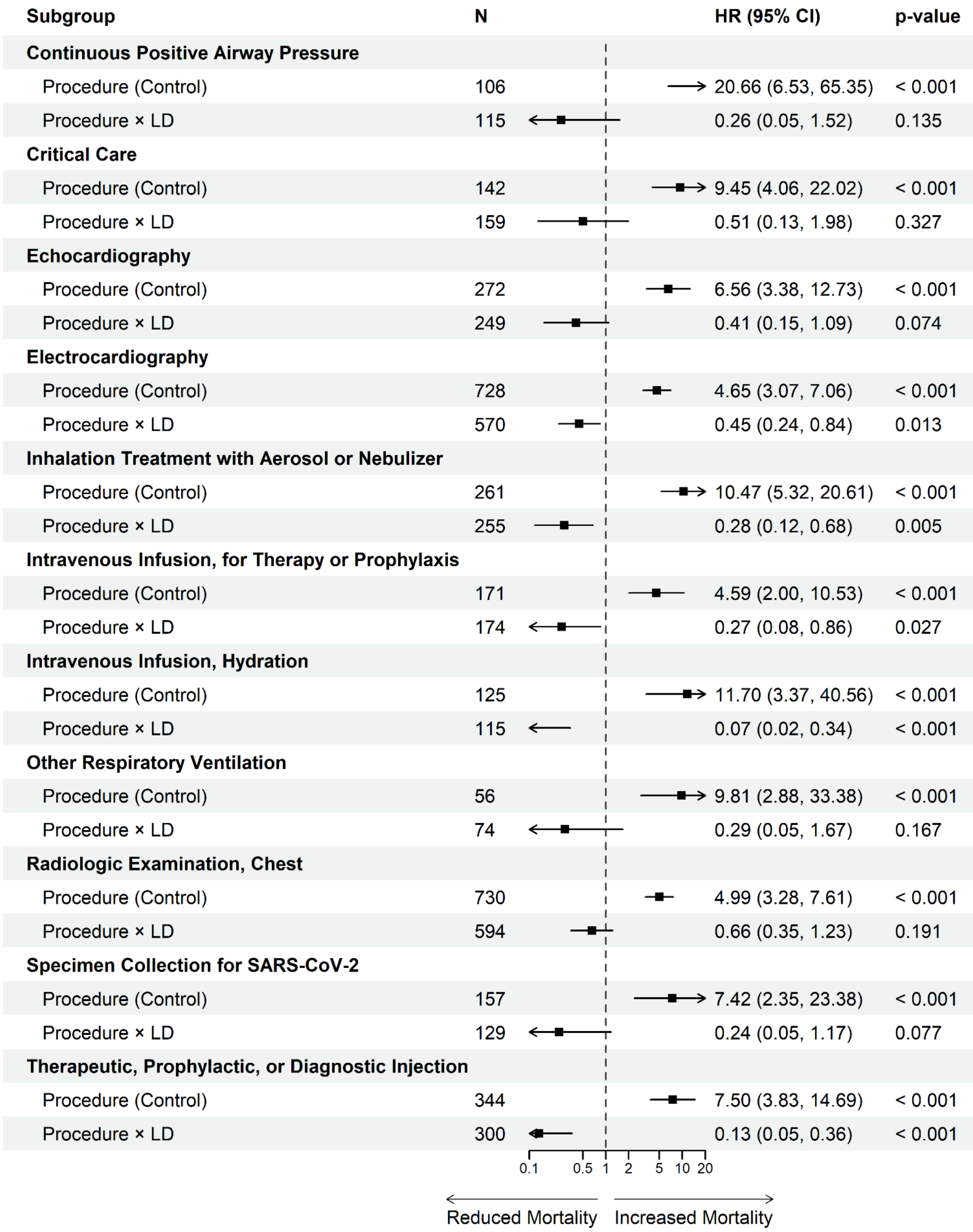

Supplementary Figure 8: Association between acute-phase procedures and mortality with LD interaction effects.

Forest plot showing hazard ratios (HR) and 95% confidence intervals from Cox proportional hazards models evaluating the association between administration of selected procedures and mortality. Estimates are shown for control patients (“Procedure (Control)”) and for the interaction term between procedure exposure and LD status (“Procedure × LD”). Models were

adjusted for Elixhauser comorbidity score and stratified by matched subclass. The vertical dashed line indicates HR = 1. Sample sizes (N) are displayed; for “Procedure × LD,” N reflects the number of patients with LDs receiving the procedure and does not necessarily correspond to the effective sample size used in model estimation. Corresponding p-values are shown.

Supplementary Tables

MONDO Parent ID	MONDO Parent Label	SNOMED ID	SNOMED Label
MONDO:0019245	lysosomal lipid storage disorder	SCTID:37312166	ATPase cation transporting 13A2 related juvenile neuronal ceroid lipofuscinosis
MONDO:0019245	lysosomal lipid storage disorder	SCTID:4047345	Acute neuronopathic Gaucher's disease
MONDO:0019245	lysosomal lipid storage disorder	SCTID:4029116	Adult GM1 gangliosidosis
MONDO:0019245	lysosomal lipid storage disorder	SCTID:4175778	Adult chronic GM2 gangliosidosis
MONDO:0019245	lysosomal lipid storage disorder	SCTID:4265641	Adult neuronal ceroid lipofuscinosis
MONDO:0019245	lysosomal lipid storage disorder	SCTID:4029879	Arylsulfatase A deficiency
MONDO:0019245	lysosomal lipid storage disorder	SCTID:608054	Atypical Gaucher disease due to saposin C deficiency
MONDO:0019245	lysosomal lipid storage disorder	SCTID:37111243	Autosomal dominant myoglobinuria
MONDO:0019245	lysosomal lipid storage disorder	SCTID:35622036	Autosomal recessive cerebellar ataxia with late-onset spasticity
MONDO:0019245	lysosomal lipid storage disorder	SCTID:4104697	B variant hexosaminidase A deficiency
MONDO:0019245	lysosomal lipid storage disorder	SCTID:4079872	B variant hexosaminidase A deficiency - adult
MONDO:0019245	lysosomal lipid storage disorder	SCTID:4029878	B variant hexosaminidase A deficiency - infantile
MONDO:0019245	lysosomal lipid storage disorder	SCTID:4031809	B variant hexosaminidase A deficiency - juvenile
MONDO:0019245	lysosomal lipid storage disorder	SCTID:4029115	B1 variant hexosaminidase A deficiency
MONDO:0019245	lysosomal lipid storage disorder	SCTID:374906	Cerebral lipidosis
MONDO:0019245	lysosomal lipid storage disorder	SCTID:4006310	Chemically-induced lipidosis
MONDO:0019245	lysosomal lipid storage disorder	SCTID:4210997	Cholesterol ester storage disease
MONDO:0019245	lysosomal lipid storage disorder	SCTID:4265894	Chronic non-neuropathic Gaucher's disease
MONDO:0019245	lysosomal lipid storage disorder	SCTID:36715284	Congenital neuronal ceroid lipofuscinosis
MONDO:0019245	lysosomal lipid storage disorder	SCTID:37166936	Dystonia due to metachromatic leucodystrophy
MONDO:0019245	lysosomal lipid storage disorder	SCTID:36715313	Encephalopathy due to prosaposin deficiency
MONDO:0019245	lysosomal lipid storage disorder	SCTID:4042934	Fabry's disease
MONDO:0019245	lysosomal lipid storage disorder	SCTID:4079873	GM1 gangliosidosis
MONDO:0019245	lysosomal lipid storage disorder	SCTID:4142635	GM2 gangliosidosis
MONDO:0019245	lysosomal lipid storage disorder	SCTID:4029117	Galactocerebroside beta-galactosidase deficiency - early onset
MONDO:0019245	lysosomal lipid storage disorder	SCTID:4105341	Galactosylceramide beta-galactosidase deficiency
MONDO:0019245	lysosomal lipid storage disorder	SCTID:4180066	Gangliosidosis
MONDO:0019245	lysosomal lipid storage disorder	SCTID:608071	Gaucher disease with ophthalmoplegia and cardiovascular calcification
MONDO:0019245	lysosomal lipid storage disorder	SCTID:4101305	Gaucher's disease
MONDO:0019245	lysosomal lipid storage disorder	SCTID:4294298	Genetic disorder of lipid storage
MONDO:0019245	lysosomal lipid storage disorder	SCTID:37396784	Genetic recurrent myoglobinuria
MONDO:0019245	lysosomal lipid storage disorder	SCTID:4165746	Globoid cell leukodystrophy, late-onset
MONDO:0019245	lysosomal lipid storage disorder	SCTID:4322172	I-cell disease
MONDO:0019245	lysosomal lipid storage disorder	SCTID:4031810	Infantile GM1 gangliosidosis
MONDO:0019245	lysosomal lipid storage disorder	SCTID:4266669	Infantile GM2 gangliosidosis
MONDO:0019245	lysosomal lipid storage disorder	SCTID:4241463	Infantile neuronal ceroid lipofuscinosis
MONDO:0019245	lysosomal lipid storage disorder	SCTID:4077298	Juvenile GM1 gangliosidosis
MONDO:0019245	lysosomal lipid storage disorder	SCTID:4318385	Juvenile GM2 gangliosidosis
MONDO:0019245	lysosomal lipid storage disorder	SCTID:4264152	Juvenile neuronal ceroid lipofuscinosis
MONDO:0019245	lysosomal lipid storage disorder	SCTID:4033084	Late-infantile neuronal ceroid lipofuscinosis
MONDO:0019245	lysosomal lipid storage disorder	SCTID:4027782	Lipid storage disease
MONDO:0019245	lysosomal lipid storage disorder	SCTID:4345570	Lipid storage myopathy
MONDO:0019245	lysosomal lipid storage disorder	SCTID:37399482	Lysosomal acid lipase deficiency
MONDO:0019245	lysosomal lipid storage disorder	SCTID:4262609	Metachromatic leukodystrophy
MONDO:0019245	lysosomal lipid storage disorder	SCTID:4180038	Metachromatic leukodystrophy due to deficiency of cerebroside sulfatase activator
MONDO:0019245	lysosomal lipid storage disorder	SCTID:604586	Metachromatic leukodystrophy due to sphingolipid activator protein I deficiency

MONDO:0019245	lysosomal lipid storage disorder	SCTID:4308755	Metachromatic leukodystrophy without arylsulfatase deficiency
MONDO:0019245	lysosomal lipid storage disorder	SCTID:4085741	Metachromatic leukodystrophy, adult type
MONDO:0019245	lysosomal lipid storage disorder	SCTID:4238363	Metachromatic leukodystrophy, congenital type
MONDO:0019245	lysosomal lipid storage disorder	SCTID:4185906	Metachromatic leukodystrophy, juvenile type
MONDO:0019245	lysosomal lipid storage disorder	SCTID:4166222	Metachromatic leukodystrophy, late infantile type
MONDO:0019245	lysosomal lipid storage disorder	SCTID:4183021	Multiple sulfatase deficiency
MONDO:0019245	lysosomal lipid storage disorder	SCTID:4170931	Neuronal ceroid lipofuscinosis
MONDO:0019245	lysosomal lipid storage disorder	SCTID:45771339	Neuronal ceroid lipofuscinosis 8
MONDO:0019245	lysosomal lipid storage disorder	SCTID:44783251	Neutral lipid storage disease with myopathy
MONDO:0019245	lysosomal lipid storage disorder	SCTID:4181427	Niemann-Pick disease, type A
MONDO:0019245	lysosomal lipid storage disorder	SCTID:4214475	Niemann-Pick disease, type B
MONDO:0019245	lysosomal lipid storage disorder	SCTID:4283682	Niemann-Pick disease, type C
MONDO:0019245	lysosomal lipid storage disorder	SCTID:4314140	Niemann-Pick disease, type C, acute form
MONDO:0019245	lysosomal lipid storage disorder	SCTID:4219251	Niemann-Pick disease, type C, chronic form
MONDO:0019245	lysosomal lipid storage disorder	SCTID:4283951	Niemann-Pick disease, type C, subacute form
MONDO:0019245	lysosomal lipid storage disorder	SCTID:4078073	Niemann-Pick disease, type D
MONDO:0019245	lysosomal lipid storage disorder	SCTID:3663236	Perinatal lethal Gaucher disease
MONDO:0019245	lysosomal lipid storage disorder	SCTID:37168357	Primary triglyceride deposit cardiomyovasculopathy
MONDO:0019245	lysosomal lipid storage disorder	SCTID:37204409	Progressive myoclonic epilepsy type 3
MONDO:0019245	lysosomal lipid storage disorder	SCTID:4119434	Pulmonary lipid storage disease
MONDO:0019245	lysosomal lipid storage disorder	SCTID:439689	Retinal dystrophy in cerebroretinal lipidosis
MONDO:0019245	lysosomal lipid storage disorder	SCTID:443895	Retinal dystrophy in systemic lipidosis
MONDO:0019245	lysosomal lipid storage disorder	SCTID:4314414	Salla disease
MONDO:0019245	lysosomal lipid storage disorder	SCTID:4031489	Sandhoff disease
MONDO:0019245	lysosomal lipid storage disorder	SCTID:4286511	Sphingolipid activator protein 1 deficiency
MONDO:0019245	lysosomal lipid storage disorder	SCTID:4079874	Sphingolipidosis
MONDO:0019245	lysosomal lipid storage disorder	SCTID:4242275	Sphingomyelin/cholesterol lipidosis
MONDO:0019245	lysosomal lipid storage disorder	SCTID:4242740	Subacute neuronopathic Gaucher's disease
MONDO:0019245	lysosomal lipid storage disorder	SCTID:4009170	Tay-Sachs disease
MONDO:0019245	lysosomal lipid storage disorder	SCTID:4324845	Tay-Sachs disease, variant AB
MONDO:0019245	lysosomal lipid storage disorder	SCTID:4029114	Total hexosaminidase deficiency - adult
MONDO:0019245	lysosomal lipid storage disorder	SCTID:4029113	Total hexosaminidase deficiency - infantile
MONDO:0019245	lysosomal lipid storage disorder	SCTID:4029752	Total hexosaminidase deficiency - juvenile
MONDO:0019245	lysosomal lipid storage disorder	SCTID:4024103	Triglyceride storage disease with ichthyosis
MONDO:0019245	lysosomal lipid storage disorder	SCTID:4221254	Wolman's disease
MONDO:0019245	lysosomal lipid storage disorder	SCTID:4029257	Xanthomatosis, familial
MONDO:0019255	sphingolipidosis	SCTID:4047345	Acute neuronopathic Gaucher's disease
MONDO:0019255	sphingolipidosis	SCTID:4029116	Adult GM1 gangliosidosis
MONDO:0019255	sphingolipidosis	SCTID:4175778	Adult chronic GM2 gangliosidosis
MONDO:0019255	sphingolipidosis	SCTID:4029879	Arylsulfatase A deficiency
MONDO:0019255	sphingolipidosis	SCTID:608054	Atypical Gaucher disease due to saposin C deficiency
MONDO:0019255	sphingolipidosis	SCTID:35622036	Autosomal recessive cerebellar ataxia with late-onset spasticity
MONDO:0019255	sphingolipidosis	SCTID:4104697	B variant hexosaminidase A deficiency
MONDO:0019255	sphingolipidosis	SCTID:4079872	B variant hexosaminidase A deficiency - adult
MONDO:0019255	sphingolipidosis	SCTID:4029878	B variant hexosaminidase A deficiency - infantile
MONDO:0019255	sphingolipidosis	SCTID:4031809	B variant hexosaminidase A deficiency - juvenile
MONDO:0019255	sphingolipidosis	SCTID:4029115	B1 variant hexosaminidase A deficiency
MONDO:0019255	sphingolipidosis	SCTID:4265894	Chronic non-neuropathic Gaucher's disease
MONDO:0019255	sphingolipidosis	SCTID:37166936	Dystonia due to metachromatic leucodystrophy
MONDO:0019255	sphingolipidosis	SCTID:36715313	Encephalopathy due to prosaposin deficiency
MONDO:0019255	sphingolipidosis	SCTID:4042934	Fabry's disease
MONDO:0019255	sphingolipidosis	SCTID:4196512	Farber's lipogranulomatosis
MONDO:0019255	sphingolipidosis	SCTID:4079873	GM1 gangliosidosis
MONDO:0019255	sphingolipidosis	SCTID:4142635	GM2 gangliosidosis
MONDO:0019255	sphingolipidosis	SCTID:4029117	Galactocerebroside beta-galactosidase deficiency - early onset

MONDO:0019255	sphingolipidosis	SCTID:4105341	Galactosylceramide beta-galactosidase deficiency
MONDO:0019255	sphingolipidosis	SCTID:4180066	Gangliosidosis
MONDO:0019255	sphingolipidosis	SCTID:608071	Gaucher disease with ophthalmoplegia and cardiovascular calcification
MONDO:0019255	sphingolipidosis	SCTID:4101305	Gaucher's disease
MONDO:0019255	sphingolipidosis	SCTID:4165746	Globoid cell leukodystrophy, late-onset
MONDO:0019255	sphingolipidosis	SCTID:4031810	Infantile GM1 gangliosidosis
MONDO:0019255	sphingolipidosis	SCTID:4266669	Infantile GM2 gangliosidosis
MONDO:0019255	sphingolipidosis	SCTID:4077298	Juvenile GM1 gangliosidosis
MONDO:0019255	sphingolipidosis	SCTID:4318385	Juvenile GM2 gangliosidosis
MONDO:0019255	sphingolipidosis	SCTID:4262609	Metachromatic leukodystrophy
MONDO:0019255	sphingolipidosis	SCTID:4180038	Metachromatic leukodystrophy due to deficiency of cerebroside sulfatase activator
MONDO:0019255	sphingolipidosis	SCTID:604586	Metachromatic leukodystrophy due to sphingolipid activator protein I deficiency
MONDO:0019255	sphingolipidosis	SCTID:4308755	Metachromatic leukodystrophy without arylsulfatase deficiency
MONDO:0019255	sphingolipidosis	SCTID:4085741	Metachromatic leukodystrophy, adult type
MONDO:0019255	sphingolipidosis	SCTID:4238363	Metachromatic leukodystrophy, congenital type
MONDO:0019255	sphingolipidosis	SCTID:4185906	Metachromatic leukodystrophy, juvenile type
MONDO:0019255	sphingolipidosis	SCTID:4166222	Metachromatic leukodystrophy, late infantile type
MONDO:0019255	sphingolipidosis	SCTID:4183021	Multiple sulfatase deficiency
MONDO:0019255	sphingolipidosis	SCTID:4181427	Niemann-Pick disease, type A
MONDO:0019255	sphingolipidosis	SCTID:4214475	Niemann-Pick disease, type B
MONDO:0019255	sphingolipidosis	SCTID:4283682	Niemann-Pick disease, type C
MONDO:0019255	sphingolipidosis	SCTID:4314140	Niemann-Pick disease, type C, acute form
MONDO:0019255	sphingolipidosis	SCTID:4219251	Niemann-Pick disease, type C, chronic form
MONDO:0019255	sphingolipidosis	SCTID:4283951	Niemann-Pick disease, type C, subacute form
MONDO:0019255	sphingolipidosis	SCTID:4078073	Niemann-Pick disease, type D
MONDO:0019255	sphingolipidosis	SCTID:3663236	Perinatal lethal Gaucher disease
MONDO:0019255	sphingolipidosis	SCTID:4031489	Sandhoff disease
MONDO:0019255	sphingolipidosis	SCTID:4286511	Sphingolipid activator protein 1 deficiency
MONDO:0019255	sphingolipidosis	SCTID:4079874	Sphingolipidosis
MONDO:0019255	sphingolipidosis	SCTID:4242275	Sphingomyelin/cholesterol lipidosis
MONDO:0019255	sphingolipidosis	SCTID:4242740	Subacute neuronopathic Gaucher's disease
MONDO:0019255	sphingolipidosis	SCTID:4009170	Tay-Sachs disease
MONDO:0019255	sphingolipidosis	SCTID:4324845	Tay-Sachs disease, variant AB
MONDO:0019255	sphingolipidosis	SCTID:4029114	Total hexosaminidase deficiency - adult
MONDO:0019255	sphingolipidosis	SCTID:4029113	Total hexosaminidase deficiency - infantile
MONDO:0019255	sphingolipidosis	SCTID:4029752	Total hexosaminidase deficiency - juvenile
MONDO:0010526	Fabry disease	SCTID:4042934	Fabry's disease
MONDO:0019249	mucopolysaccharidosis	SCTID:4199300	Deficiency of N-acetylgalactosamine-4-sulfatase
MONDO:0019249	mucopolysaccharidosis	SCTID:4208985	Hunter's syndrome, mild form
MONDO:0019249	mucopolysaccharidosis	SCTID:4247774	Hunter's syndrome, severe form
MONDO:0019249	mucopolysaccharidosis	SCTID:4287262	Maroteaux-Lamy syndrome
MONDO:0019249	mucopolysaccharidosis	SCTID:4094220	Maroteaux-Lamy syndrome, intermediate form
MONDO:0019249	mucopolysaccharidosis	SCTID:4285890	Maroteaux-Lamy syndrome, mild form
MONDO:0019249	mucopolysaccharidosis	SCTID:4238503	Maroteaux-Lamy syndrome, severe form
MONDO:0019249	mucopolysaccharidosis	SCTID:4292105	Morquio syndrome
MONDO:0019249	mucopolysaccharidosis	SCTID:433446	Mucopolysaccharidosis
MONDO:0019249	mucopolysaccharidosis	SCTID:4294142	Mucopolysaccharidosis, MPS-I
MONDO:0019249	mucopolysaccharidosis	SCTID:4276347	Mucopolysaccharidosis, MPS-I-H
MONDO:0019249	mucopolysaccharidosis	SCTID:4097554	Mucopolysaccharidosis, MPS-I-H/S
MONDO:0019249	mucopolysaccharidosis	SCTID:4218929	Mucopolysaccharidosis, MPS-I-S
MONDO:0019249	mucopolysaccharidosis	SCTID:4323827	Mucopolysaccharidosis, MPS-II
MONDO:0019249	mucopolysaccharidosis	SCTID:4215209	Mucopolysaccharidosis, MPS-III-A
MONDO:0019249	mucopolysaccharidosis	SCTID:4243053	Mucopolysaccharidosis, MPS-III-B
MONDO:0019249	mucopolysaccharidosis	SCTID:4326882	Mucopolysaccharidosis, MPS-III-C

MONDO:0019249	mucopolysaccharidosis	SCTID:4049596	Mucopolysaccharidosis, MPS-III-D
MONDO:0019249	mucopolysaccharidosis	SCTID:4219277	Mucopolysaccharidosis, MPS-IV-A
MONDO:0019249	mucopolysaccharidosis	SCTID:4031815	Mucopolysaccharidosis, MPS-IV-B
MONDO:0019249	mucopolysaccharidosis	SCTID:4184782	Mucopolysaccharidosis, MPS-VII
MONDO:0019249	mucopolysaccharidosis	SCTID:37162245	Mucopolysaccharidosis-like plus disease
MONDO:0019249	mucopolysaccharidosis	SCTID:4227905	Sanfilippo syndrome
MONDO:0018868	metachromatic leukodystrophy	SCTID:4029879	Arylsulfatase A deficiency
MONDO:0018868	metachromatic leukodystrophy	SCTID:4280234	Deficiency of cerebroside-sulfatase
MONDO:0018868	metachromatic leukodystrophy	SCTID:37166936	Dystonia due to metachromatic leucodystrophy
MONDO:0018868	metachromatic leukodystrophy	SCTID:4262609	Metachromatic leukodystrophy
MONDO:0018868	metachromatic leukodystrophy	SCTID:4180038	Metachromatic leukodystrophy due to deficiency of cerebroside sulfatase activator
MONDO:0018868	metachromatic leukodystrophy	SCTID:604586	Metachromatic leukodystrophy due to sphingolipid activator protein I deficiency
MONDO:0018868	metachromatic leukodystrophy	SCTID:4308755	Metachromatic leukodystrophy without arylsulfatase deficiency
MONDO:0018868	metachromatic leukodystrophy	SCTID:4085741	Metachromatic leukodystrophy, adult type
MONDO:0018868	metachromatic leukodystrophy	SCTID:4238363	Metachromatic leukodystrophy, congenital type
MONDO:0018868	metachromatic leukodystrophy	SCTID:4185906	Metachromatic leukodystrophy, juvenile type
MONDO:0018868	metachromatic leukodystrophy	SCTID:4166222	Metachromatic leukodystrophy, late infantile type
MONDO:0018868	metachromatic leukodystrophy	SCTID:4286511	Sphingolipid activator protein 1 deficiency
MONDO:0018150	Gaucher disease	SCTID:4047345	Acute neuronopathic Gaucher's disease
MONDO:0018150	Gaucher disease	SCTID:608054	Atypical Gaucher disease due to saposin C deficiency
MONDO:0018150	Gaucher disease	SCTID:4265894	Chronic non-neuropathic Gaucher's disease
MONDO:0018150	Gaucher disease	SCTID:608071	Gaucher disease with ophthalmoplegia and cardiovascular calcification
MONDO:0018150	Gaucher disease	SCTID:4101305	Gaucher's disease
MONDO:0018150	Gaucher disease	SCTID:3663236	Perinatal lethal Gaucher disease
MONDO:0018150	Gaucher disease	SCTID:4242740	Subacute neuronopathic Gaucher's disease
MONDO:0016295	neuronal ceroid lipofuscinosis	SCTID:37312166	ATPase cation transporting 13A2 related juvenile neuronal ceroid lipofuscinosis
MONDO:0016295	neuronal ceroid lipofuscinosis	SCTID:4265641	Adult neuronal ceroid lipofuscinosis
MONDO:0016295	neuronal ceroid lipofuscinosis	SCTID:36715284	Congenital neuronal ceroid lipofuscinosis
MONDO:0016295	neuronal ceroid lipofuscinosis	SCTID:4241463	Infantile neuronal ceroid lipofuscinosis
MONDO:0016295	neuronal ceroid lipofuscinosis	SCTID:4264152	Juvenile neuronal ceroid lipofuscinosis
MONDO:0016295	neuronal ceroid lipofuscinosis	SCTID:4033084	Late-infantile neuronal ceroid lipofuscinosis
MONDO:0016295	neuronal ceroid lipofuscinosis	SCTID:4170931	Neuronal ceroid lipofuscinosis
MONDO:0016295	neuronal ceroid lipofuscinosis	SCTID:45771339	Neuronal ceroid lipofuscinosis 8
MONDO:0016295	neuronal ceroid lipofuscinosis	SCTID:37204409	Progressive myoclonic epilepsy type 3
MONDO:0017719	gangliosidosis	SCTID:4029116	Adult GM1 gangliosidosis
MONDO:0017719	gangliosidosis	SCTID:4175778	Adult chronic GM2 gangliosidosis
MONDO:0017719	gangliosidosis	SCTID:4104697	B variant hexosaminidase A deficiency
MONDO:0017719	gangliosidosis	SCTID:4079872	B variant hexosaminidase A deficiency - adult
MONDO:0017719	gangliosidosis	SCTID:4029878	B variant hexosaminidase A deficiency - infantile
MONDO:0017719	gangliosidosis	SCTID:4031809	B variant hexosaminidase A deficiency - juvenile
MONDO:0017719	gangliosidosis	SCTID:4029115	B1 variant hexosaminidase A deficiency
MONDO:0017719	gangliosidosis	SCTID:4079873	GM1 gangliosidosis
MONDO:0017719	gangliosidosis	SCTID:4142635	GM2 gangliosidosis
MONDO:0017719	gangliosidosis	SCTID:4180066	Gangliosidosis
MONDO:0017719	gangliosidosis	SCTID:4031810	Infantile GM1 gangliosidosis
MONDO:0017719	gangliosidosis	SCTID:4266669	Infantile GM2 gangliosidosis
MONDO:0017719	gangliosidosis	SCTID:4077298	Juvenile GM1 gangliosidosis
MONDO:0017719	gangliosidosis	SCTID:4318385	Juvenile GM2 gangliosidosis
MONDO:0017719	gangliosidosis	SCTID:4031489	Sandhoff disease
MONDO:0017719	gangliosidosis	SCTID:4009170	Tay-Sachs disease
MONDO:0017719	gangliosidosis	SCTID:4324845	Tay-Sachs disease, variant AB
MONDO:0017719	gangliosidosis	SCTID:4029114	Total hexosaminidase deficiency - adult
MONDO:0017719	gangliosidosis	SCTID:4029113	Total hexosaminidase deficiency - infantile

MONDO:0017719	gangliosidosis	SCTID:4029752	Total hexosaminidase deficiency - juvenile
MONDO:0019246	inborn disorder of lysosomal amino acid transport	SCTID:44809321	Adult cystinosis
MONDO:0019246	inborn disorder of lysosomal amino acid transport	SCTID:4094164	Benign adult cystinosis
MONDO:0019246	inborn disorder of lysosomal amino acid transport	SCTID:4126105	Congenital Fanconi syndrome
MONDO:0019246	inborn disorder of lysosomal amino acid transport	SCTID:4100827	Cystinosis
MONDO:0019246	inborn disorder of lysosomal amino acid transport	SCTID:4079879	Disorder of sialic acid metabolism
MONDO:0019246	inborn disorder of lysosomal amino acid transport	SCTID:4268774	Infantile nephropathic cystinosis
MONDO:0019246	inborn disorder of lysosomal amino acid transport	SCTID:4027350	Juvenile nephropathic cystinosis
MONDO:0019246	inborn disorder of lysosomal amino acid transport	SCTID:4314414	Salla disease
MONDO:0019246	inborn disorder of lysosomal amino acid transport	SCTID:4146367	Sialic acid storage disease, severe infantile type
MONDO:0019246	inborn disorder of lysosomal amino acid transport	SCTID:4150750	Sialic storage disease
MONDO:0019246	inborn disorder of lysosomal amino acid transport	SCTID:4031943	Sialuria
MONDO:0016239	cystinosis	SCTID:44809321	Adult cystinosis
MONDO:0016239	cystinosis	SCTID:4094164	Benign adult cystinosis
MONDO:0016239	cystinosis	SCTID:4126105	Congenital Fanconi syndrome
MONDO:0016239	cystinosis	SCTID:4100827	Cystinosis
MONDO:0016239	cystinosis	SCTID:4268774	Infantile nephropathic cystinosis
MONDO:0016239	cystinosis	SCTID:4027350	Juvenile nephropathic cystinosis
MONDO:0017731	glycoproteinosis	SCTID:4163879	Adult fucosidosis
MONDO:0017731	glycoproteinosis	SCTID:4029122	Alpha-N-acetylgalactosaminidase deficiency
MONDO:0017731	glycoproteinosis	SCTID:604334	Alpha-N-acetylgalactosaminidase deficiency type 1
MONDO:0017731	glycoproteinosis	SCTID:619007	Alpha-N-acetylgalactosaminidase deficiency type 2
MONDO:0017731	glycoproteinosis	SCTID:619008	Alpha-N-acetylgalactosaminidase deficiency type 3
MONDO:0017731	glycoproteinosis	SCTID:4207521	Aspartylglucosaminuria
MONDO:0017731	glycoproteinosis	SCTID:4031942	Beta-D-mannosidosis
MONDO:0017731	glycoproteinosis	SCTID:4262271	Combined deficiency of sialidase AND beta galactosidase
MONDO:0017731	glycoproteinosis	SCTID:4181428	Dysmorphic sialidosis
MONDO:0017731	glycoproteinosis	SCTID:4217611	Dysmorphic sialidosis with renal involvement
MONDO:0017731	glycoproteinosis	SCTID:4100490	Dysmorphic sialidosis, congenital form
MONDO:0017731	glycoproteinosis	SCTID:4202220	Dysmorphic sialidosis, infantile form
MONDO:0017731	glycoproteinosis	SCTID:4008557	Dysmorphic sialidosis, juvenile form
MONDO:0017731	glycoproteinosis	SCTID:4277436	Fucosidosis
MONDO:0017731	glycoproteinosis	SCTID:4299945	Glycoprotein storage disorder
MONDO:0017731	glycoproteinosis	SCTID:4322172	I-cell disease
MONDO:0017731	glycoproteinosis	SCTID:4246880	Infantile fucosidosis
MONDO:0017731	glycoproteinosis	SCTID:4161004	Juvenile fucosidosis
MONDO:0017731	glycoproteinosis	SCTID:4277255	Mannosidosis
MONDO:0017731	glycoproteinosis	SCTID:4266511	Mannosidosis, type I
MONDO:0017731	glycoproteinosis	SCTID:4241350	Mannosidosis, type II
MONDO:0017731	glycoproteinosis	SCTID:4323268	Mucolipidosis
MONDO:0017731	glycoproteinosis	SCTID:37110837	Mucolipidosis type IV
MONDO:0017731	glycoproteinosis	SCTID:607719	Oligosaccharidosis
MONDO:0017731	glycoproteinosis	SCTID:4278525	Pseudo-Hurler polydystrophy
MONDO:0017731	glycoproteinosis	SCTID:4307744	Sialidosis
MONDO:0017731	glycoproteinosis	SCTID:37109814	Sialidosis type 1
unassigned	unassigned	SCTID:4212254	Acid phosphatase deficiency

unassigned	unassigned	SCTID:4185398	Arylsulfatase deficiency without MLD (metachromatic leukodystrophy)
unassigned	unassigned	SCTID:4167868	Danon disease
unassigned	unassigned	SCTID:4053270	Disorder of lysosomal enzyme
unassigned	unassigned	SCTID:4109172	Dysostosis multiplex
unassigned	unassigned	SCTID:4150135	Dysostosis multiplex group
unassigned	unassigned	SCTID:4151377	Intestinal lipofuscinosis
unassigned	unassigned	SCTID:4030564	Lipofuscinosis
unassigned	unassigned	SCTID:37155637	Lysosomal storage disease
unassigned	unassigned	SCTID:4302604	Pancreatic triacylglycerol lipase deficiency

Supplementary Table 1: Mapping of SNOMED diagnoses to MONDO parent categories. SNOMED-coded lysosomal disorder diagnoses were mapped to higher-level MONDO parent categories based on hierarchical relationships in the Mondo Disease Ontology; assignments represent parent-child relationships and not necessarily exact synonyms or cross-references.

Mondo ID	Mondo Label	Patient Count
MONDO:0019245	lysosomal lipid storage disorder	14654
MONDO:0019255	sphingolipidosis	7774
MONDO:0010526	Fabry disease	1117
MONDO:0019249	mucopolysaccharidosis	1096
MONDO:0018868	metachromatic leukodystrophy	467
MONDO:0018150	Gaucher disease	455
MONDO:0016295	neuronal ceroid lipofuscinosis	303
MONDO:0017719	gangliosidosis	298
MONDO:0019246	inborn disorder of lysosomal amino acid transport	240
MONDO:0016239	cystinosis	238
MONDO:0017731	glycoproteinosis	30

Supplementary Table 2: Lysosomal Disorder Rolled-Up Category Counts in the Baseline Matched Cohort.

Mondo ID	Mondo Label	Patient Count
MONDO:0019245	lysosomal lipid storage disorder	5046
MONDO:0019255	sphingolipidosis	2624
MONDO:0010526	Fabry disease	368
MONDO:0019249	mucopolysaccharidosis	321
MONDO:0018868	metachromatic leukodystrophy	160
MONDO:0018150	Gaucher disease	145
MONDO:0017719	gangliosidosis	105
MONDO:0016295	neuronal ceroid lipofuscinosis	99
MONDO:0019246	inborn disorder of lysosomal amino acid transport	76
MONDO:0016239	cystinosis	76
MONDO:0017731	glycoproteinosis	<20

Supplementary Table 3: Lysosomal Disorder Rolled-Up Category Counts in the COVID Matched Cohort.

Codeset ID	Concept Set Name
270102919	[Pitt N3C] Heparin Sodium (UFH)
932266800	systemic hydrocortisone35
231875380	famotidine-105
425433230	[DM]Insulin
1000082442	Nirmatrelvir, All Descendants
769518525	budesonide
883259145	albuterol
1000011148	Dexamethasone--non otic, ophthalm, MDI

1000033171	N3C Lisinopril
56320741	aspirin
745170047	[CVDT] Carvedilol
940495880	[CVDT] Methylprednisolone
65058498	[Pitt N3C] Prednisone
472540083	apixaban
1000040403	Remdesivir, All Descendants

Supplementary Table 4: N3C codeset identifiers and concept set names for 15 drugs administered in the acute COVID window.

Codeset ID	Concept Set Name
577359343	echocardiography procedures
30670147	electrocardiography procedures
920228667	radiologic examination, chest
209464212	intravenous infusion, for therapy or prophylaxis
996569760	continuous positive airway pressure
5668392	other respiratory ventilation
993408479	Specimen collection for SARS-CoV-2
147612319	therapeutic, prophylactic, or diagnostic injection
970208606	critical care
764129526	inhalation treatment with aerosol or nebulizer
632643495	intravenous infusion, hydration

Supplementary Table 5: N3C codeset identifiers and concept set names for 11 procedures administered in the acute COVID window.

**Characteristic**	**Control**<br>N = 32,238	**LD**<br>N = 16,119
**Clinical Factors**		
Number of Visits	54 (21, 121)	89 (39, 182)
Days Observed	1,945 (1,374, 2,241)	1,945 (1,373, 2,241)

BMI	28 (23, 33)	28 (22, 33)
COVID Positive	11,328 (35%)	5,590 (35%)
**Race**		
White	25,069 (78%)	12,536 (78%)
Black or African American	3,197 (9.9%)	1,598 (9.9%)
Asian	862 (2.7%)	431 (2.7%)
American Indian or Alaska Native	142 (0.4%)	71 (0.4%)
Native Hawaiian or Other Pacific Islander	108 (0.3%)	54 (0.3%)
Other/Unknown	2,860 (8.9%)	1,429 (8.9%)
Age	56 (33, 70)	56 (34, 70)
**Sex**		
Female	17,358 (54%)	8,679 (54%)
Male	14,060 (44%)	7,030 (44%)
Unknown	820 (2.5%)	410 (2.5%)
**Comorbidities**		
Congestive Heart Failure	4,277 (13%)	3,786 (23%)
Cardiac Arrhythmias	11,241 (35%)	8,872 (55%)
Valvular Disease	4,904 (15%)	4,089 (25%)
Pulmonary Circulation Disorders	2,438 (7.6%)	2,544 (16%)
Peripheral Vascular Disorders	4,208 (13%)	3,524 (22%)
Uncomplicated Hypertension	10,032 (31%)	5,185 (32%)
Complicated Hypertension	5,171 (16%)	4,332 (27%)
Paralysis	1,036 (3.2%)	1,566 (9.7%)
Other Neurological Disorders	5,040 (16%)	5,764 (36%)

Chronic Pulmonary Disease	9,654 (30%)	6,115 (38%)
Uncomplicated Diabetes	2,286 (7.1%)	1,343 (8.3%)
Complicated Diabetes	5,177 (16%)	4,375 (27%)
Hypothyroidism	4,994 (15%)	3,095 (19%)
Renal Failure	4,301 (13%)	3,797 (24%)
Liver Disease	4,336 (13%)	3,686 (23%)
Peptic Ulcer Disease without Bleeding	902 (2.8%)	851 (5.3%)
AIDS/HIV	317 (1.0%)	278 (1.7%)
Lymphoma	477 (1.5%)	355 (2.2%)
Metastatic Cancer	1,421 (4.4%)	1,351 (8.4%)
Solid Tumor without Metastasis	2,826 (8.8%)	1,538 (9.5%)
Rheumatoid Arthritis or Collagen Vascular Disease	2,822 (8.8%)	2,552 (16%)
Coagulopathy	3,399 (11%)	3,482 (22%)
Obesity	8,884 (28%)	5,550 (34%)
Weight Loss	1,596 (5.0%)	1,991 (12%)
Fluid and Electrolyte Disorders	8,116 (25%)	6,755 (42%)
Blood Loss Anemia	923 (2.9%)	871 (5.4%)
Deficiency Anemia	3,557 (11%)	3,103 (19%)
Alcohol Abuse	5,094 (16%)	5,103 (32%)
Drug Abuse	3,220 (10.0%)	3,044 (19%)
Psychoses	1,026 (3.2%)	845 (5.2%)
Depression	9,768 (30%)	6,683 (41%)
Elixhauser Score	2 (-1, 15)	11 (0, 29)
**Region**		
Atlantic	8,106 (25%)	3,766 (23%)
Central	15,616 (48%)	8,045 (50%)
Southern	2,394 (7.4%)	1,248 (7.7%)
Western Pacific	3,567 (11%)	1,757 (11%)
Unknown	2,555 (7.9%)	1,303 (8.1%)

Supplementary Table 6: Characteristics of the Baseline Matched Cohort. Summary table for patients in the Baseline Matched Cohort, including clinical factors, demographics (age, race, sex), comorbidities, and geographic region. Discrete data are displayed as “count (percentage)” and continuous data as “median (IQR)”.

Lysosomal Disorder	N	Age	COVID Positive	Days Observed	Number of Visits	BMI	Elixhauser Score
Lipid storage disease	6,543	65 (53, 75)	2,391 (37%)	1,926 (1,372, 2,201)	83 (38, 161)	29 (24, 34)	8 (-1, 24)
Sphingolipidosis	5,191	47 (27, 66)	1,780 (34%)	2,107 (1,575, 2,305)	105 (43, 215)	28 (22, 34)	18 (5, 38)
Fabry's disease	1,117	49 (33, 63)	384 (34%)	1,863 (1,296, 2,177)	76 (31, 158)	26 (21, 31)	8 (0, 20)
Metachromatic leukodystrophy	461	49 (21, 68)	161 (35%)	2,092 (1,433, 2,318)	132 (57, 277)	27 (19, 33)	21 (10, 38)
Gaucher's disease	446	47 (31, 68)	151 (34%)	1,553 (1,014, 2,049)	56 (26, 134)	24 (21, 29)	6 (0, 19)
Mucopolysaccharidosis	402	38 (18, 64)	123 (31%)	1,830 (1,149, 2,219)	77 (34, 159)	25 (20, 31)	10 (0, 25)
MPS-I-H	330	14 (7, 30)	80 (24%)	1,825 (1,150, 2,182)	82 (40, 188)	21 (17, 27)	7 (0, 18)
Neuronal ceroid lipofuscinosis	287	47 (18, 63)	96 (33%)	1,828 (1,170, 2,178)	74 (38, 145)	25 (20, 31)	24 (10, 39)
Cystinosis	228	30 (18, 46)	76 (33%)	1,907 (1,272, 2,235)	117 (48, 254)	23 (18, 28)	13 (4, 27)
MPS-II	215	30 (15, 63)	72 (33%)	1,890 (1,289, 2,216)	90 (41, 178)	25 (21, 31)	6 (0, 24)
Tay-Sachs disease	137	39 (24, 56)	43 (31%)	1,645 (888, 2,101)	78 (33, 154)	27 (21, 32)	4 (-3, 19)
MPS-IV-A	99	24 (12, 51)	21 (21%)	1,941 (1,458, 2,212)	95 (42, 173)	25 (21, 31)	3 (0, 11)
Galactosylceramide beta-galactosidase deficiency	94	23 (7, 38)	25 (27%)	1,517 (801, 1,935)	57 (20, 105)	22 (17, 28)	10 (0, 17)
GM2 gangliosidosis	72	76 (63, 83)	31 (43%)	2,027 (1,353, 2,228)	114 (47, 210)	26 (21, 30)	18 (3, 33)
Niemann-Pick disease, type C	66	28 (16, 40)	21 (32%)	1,729 (1,145, 2,106)	82 (44, 144)	22 (17, 27)	13 (9, 30)
Gangliosidosis	65	49 (6, 66)	25 (38%)	1,714 (935, 2,043)	56 (33, 103)	21 (18, 31)	10 (0, 21)

Sphingomyelin/cholesterol lipidosis	44	37 (30, 57)	< 20 (< 45%)	1,561 (793, 2,089)	66 (32, 83)	28 (22, 33)	2 (-2, 14)
Niemann-Pick disease, type B	29	43 (29, 56)	< 20 (< 69%)	1,364 (725, 2,010)	64 (18, 172)	25 (21, 30)	13 (0, 27)
MPS-I-H/S	29	21 (6, 30)	< 20 (< 69%)	1,756 (879, 2,127)	101 (55, 205)	25 (20, 28)	10 (0, 20)
Danon disease	27	25 (20, 38)	< 20 (< 74%)	1,756 (728, 2,254)	144 (53, 218)	25 (19, 34)	20 (13, 42)
Cerebral lipidosis	24	29 (9, 55)	< 20 (< 83%)	1,775 (1,072, 2,165)	119 (85, 156)	21 (16, 27)	10 (1, 20)
Sandhoff disease	21	22 (6, 39)	< 20 (< 95%)	1,656 (1,039, 2,034)	74 (44, 131)	23 (17, 29)	8 (0, 18)
MPS-I-S	21	30 (10, 46)	< 20 (< 95%)	1,896 (1,288, 2,099)	91 (46, 163)	25 (19, 32)	2 (0, 17)
Other	171	22 (11, 47)	42 (25%)	1,600 (1,176, 2,160)	87 (37, 190)	21 (17, 27)	10 (0, 21)

Supplementary Table 7: Lysosomal disorder characteristics in the Baseline Matched Cohort.
Summary table for lysosomal disorder patients in the Baseline Matched Cohort, stratified by SNOMED lysosomal disorder subtype labels for increased granularity. “Other” contains subtypes with counts fewer than 20 patients. Discrete data are displayed as “count (percentage)” and continuous data as “median (IQR)”. Counts fewer than 20 are obscured with “< 20 (< X%)”.

**Elixhauser Comorbidity**	**Category**	**ICD-10 Prefixes**
Congestive heart failure	Cardiometabolic	I099, I110, I130, I132, I255, I420, I425, I426, I427, I428, I429, I43, I50, P290
Cardiac arrhythmias	Cardiometabolic	I441, I442, I443, I456, I459, I47, I48, I49, R000, R001, R008, T821, Z450, Z950
Valvular disease	Cardiometabolic	A520, I05, I06, I07, I08, I091, I098, I34, I35, I36, I37, I38, I39, Q230, Q231, Q232, Q233, Z952, Z953, Z954
Pulmonary circulation disorders	Cardiometabolic	I26, I27, I280, I288, I289
Peripheral vascular disorders	Cardiometabolic	I70, I71, I731, I738, I739, I771, I790, I792, K551, K558, K559, Z958, Z959
Hypertension, uncomplicated	Cardiometabolic	I10
Hypertension, complicated	Cardiometabolic	I11, I12, I13, I15
Diabetes, uncomplicated	Cardiometabolic	E100, E101, E109, E110, E111, E119, E120, E121, E129, E130, E131, E139, E140, E141, E149
Diabetes, complicated	Cardiometabolic	E102, E103, E104, E105, E106, E107, E108, E112, E113, E114, E115, E116, E117, E118, E122, E123, E124, E125, E126, E127, E128, E132, E133, E134, E135, E136, E137, E138, E142, E143, E144, E145, E146, E147, E148
Obesity	Cardiometabolic	E66
Chronic pulmonary disease	Pulmonary	I278, I279, J40, J41, J42, J43, J44, J45, J46, J47, J60, J61, J62, J63, J64, J65, J66, J67, J684, J701, J703
Renal failure	Renal	I120, I131, N18, N19, N250, Z490, Z491, Z492, Z940, Z992
Liver disease	Hepatic/GI	B18, I85, I864, I982, K70, K711, K713, K714, K715, K717, K72, K73, K74, K760, K762, K763, K764, K765, K766, K767, K768, K769, Z944
Peptic ulcer disease	Hepatic/GI	K257, K259, K267, K269, K277, K279, K287, K289
Paralysis	Neurologic	G041, G114, G801, G802, G81, G82, G830, G831, G832, G833, G834, G839
Other neurological disorders	Neurologic	G10, G11, G12, G13, G20, G21, G22, G254, G255, G312, G318, G319, G32, G35, G36, G37, G40, G41, G931, G934, R470, R56

Hypothyroidism	Endocrine	E00, E01, E02, E03, E890
Coagulopathy	Hematologic	D65, D66, D67, D68, D691, D693, D694, D695, D696
Blood loss anemia	Hematologic	D500
Deficiency anemia	Hematologic	D508, D509, D51, D52, D53
Weight loss	Nutritional	E40, E41, E42, E43, E44, E45, E46, R634, R64
Fluid and electrolyte disorders	Nutritional	E222, E86, E87
Lymphoma	Malignancy	C81, C82, C83, C84, C85, C88, C96, C900, C902
Metastatic cancer	Malignancy	C77, C78, C79, C80
Solid tumor, without metastasis	Malignancy	C00–C97
Collagen vascular disease	Autoimmune	L940, L941, L943, M05, M06, M08, M120, M123, M30, M310, M311, M312, M313, M32, M33, M34, M35, M45, M461, M468, M469
AIDS/HIV	Infectious	B20, B21, B22, B24
Alcohol abuse	Substance Use	F10, E52, G621, I426, K292, K700, K703, K709, T51, Z502, Z714, Z721
Drug abuse	Substance Use	F11, F12, F13, F14, F15, F16, F18, F19, Z715, Z722
Psychoses	Psychiatric	F20, F22, F23, F24, F25, F28, F29, F302, F312, F315
Depression	Psychiatric	F204, F313, F314, F315, F32, F33, F341, F412, F432

Supplementary Table 8: Mapping of Elixhauser comorbidities to organ system categories and ICD-10 prefixes.
Comorbidities were defined using ICD-10 code prefixes and were mapped to organ system categories.

Severity	Median Δ	Mean Δ	Max Δ
Mild No ED	7.95302	7.43935	20.1585
Mild ED	11.9213	11.9335	30.8186
Moderate	8.93014	8.82326	23.2213
Severe	6.93816	7.73853	19.0799
Death	6.00414	7.91564	19.9275

Supplementary Table 9: Differences in comorbidity distributions across severity strata.
Δ is defined as the difference in percentage prevalence between the LD and control groups (Δ = %LD − %control). Summary statistics (median, mean, and maximum Δ) are reported for each severity stratum.

LD Category	OR (95% CI)	p-value	FDR q-value
Lysosomal disorder (Overall)	0.98 (0.94, 1.02)	0.287	0.788
Sphingolipidosis	0.95 (0.89, 1.01)	0.082	0.637
Lysosomal lipid storage disorder	1.00 (0.96, 1.05)	0.994	0.994
Fabry disease	1.02 (0.86, 1.20)	0.822	0.994
Gaucher disease	1.12 (0.87, 1.45)	0.364	0.800
Metachromatic leukodystrophy	0.85 (0.67, 1.08)	0.174	0.637
Inborn disorder of lysosomal amino acid transport	0.95 (0.67, 1.34)	0.771	0.994
Gangliosidosis	1.28 (0.93, 1.77)	0.125	0.637
Cystinosis	0.98 (0.69, 1.38)	0.907	0.994
Neuronal ceroid lipofuscinosis	1.02 (0.75, 1.39)	0.875	0.994
Glycoproteinosis	0.91 (0.32, 2.60)	0.860	0.994

Supplementary Table 10: Conditional logistic regression for SARS-CoV-2 infection in the Baseline Matched Cohort by lysosomal disorder category.
Odds ratios (OR) for SARS-CoV-2 infection. Models were conditioned on matching variables (age, sex, race, days observed, and data contributing site). "—" indicates estimates not estimated; mucopolysaccharidosis was not estimated due to dataset limitations.

LD Category	Comorbidity Covariates
Sphingolipidosis	Cardiac arrhythmias, Hypertension (uncomplicated), Paralysis, Other neurological disorders, Diabetes (uncomplicated), Diabetes (complicated), Liver disease, Collagen vascular disease, Weight loss, Fluid and electrolyte disorders, Alcohol abuse
Lysosomal lipid storage disorder	Cardiac arrhythmias, Paralysis, Other neurological disorders, Diabetes (complicated), Liver disease, Collagen vascular disease, Weight loss, Deficiency anemia, Alcohol abuse
Fabry disease	Valvular disease, Renal failure
Gaucher disease	Coagulopathy
Metachromatic leukodystrophy	Paralysis, Other neurological disorders
Gangliosidosis	Other neurological disorders
Neuronal ceroid lipofuscinosis	Other neurological disorders

Supplementary Table 11: Comorbidity covariates included in adjusted models by lysosomal disorder (LD) category.
Covariates were selected using category-specific logistic regression analyses ($p < 0.001$), regardless of the direction of association, and were included as covariates in adjusted conditional logistic regression models.

Comparison	Group	N at risk	Events	Survival probability (95% CI)
Overall: LD vs Control	LD	5,106	264	0.952 (0.946, 0.957)
Overall: LD vs Control	Control	10,306	328	0.970 (0.967, 0.973)
LD: Hospitalized vs Non-Hospitalized	Hospitalized	937	209	0.821 (0.799, 0.843)
LD: Hospitalized vs Non-Hospitalized	Non-Hospitalized	4,169	55	0.987 (0.984, 0.991)
Control: Hospitalized vs Non-Hospitalized	Hospitalized	1,161	264	0.820 (0.800, 0.840)
Control: Hospitalized vs Non-Hospitalized	Non-Hospitalized	9,145	64	0.993 (0.992, 0.995)

Supplementary Table 12: One-year survival probabilities stratified by lysosomal disorder (LD) status and hospitalization.
Survival probabilities are presented as Kaplan–Meier estimates with 95% confidence intervals (CI). N at risk and cumulative events are shown for each group.

Reporting Summary

The RECORD statement – checklist of items, extended from the STROBE statement, that should be reported in observational studies using routinely collected health data.

	Item No.	STROBE items	Location in manuscript where items are reported	RECORD items	Location in manuscript where items are reported
**Title and abstract**					
	1	(a) Indicate the study's design with a commonly used term in the title or the abstract<br>(b) Provide in the abstract an informative and balanced summary of what was done and what was found	Abstract	RECORD 1.1: The type of data used should be specified in the title or abstract. When possible, the name of the databases used should be included.<br><br>RECORD 1.2: If applicable, the geographic region and timeframe within which the study took place should be reported in the title or abstract.<br><br>RECORD 1.3: If linkage between databases was conducted for the study, this should be clearly stated in the title or abstract.	Abstract ("retrospective cohort study… electronic health record data… National Clinical Cohort Collaborative (N3C)")<br><br>Abstract ("nationwide U.S…. longitudinal records starting January 1, 2018… captured between January 1, 2020, and July 11, 2024")<br><br>N/A (single centralized database, no linkage)
**Introduction**					
Background rationale	2	Explain the scientific background and rationale for the investigation being reported	Abstract; Background		
Objectives	3	State specific objectives, including any prespecified hypotheses	Background (final paragraph)		
**Methods**					
Study Design	4	Present key elements of study design early in the paper	Methods (Data Source & Preprocessing)		

Setting	5	Describe the setting, locations, and relevant dates, including periods of recruitment, exposure, follow-up, and data collection	Methods (Data Source & Key Definitions)		
Participants	6	*(a) Cohort study* - Give the eligibility criteria, and the sources and methods of selection of participants. Describe methods of follow-up<br><br>*(b) Cohort study* - For matched studies, give matching criteria and number of exposed and unexposed		RECORD 6.1: The methods of study population selection (such as codes or algorithms used to identify subjects) should be listed in detail. If this is not possible, an explanation should be provided.<br><br>RECORD 6.2: Any validation studies of the codes or algorithms used to select the population should be referenced. If validation was conducted for this study and not published elsewhere, detailed methods and results should be provided.<br><br>RECORD 6.3: If the study involved linkage of databases, consider use of a flow diagram or other graphical display to demonstrate the data linkage process, including the number of individuals with linked data at each stage.	Methods (Key Definitions, Patient Identification and Classification, Survival analysis)<br><br>Methods (Patient Identification and Classification)<br><br>N/A (single centralized database, no linkage)
Variables	7	Clearly define all outcomes, exposures, predictors, potential confounders, and effect modifiers. Give diagnostic criteria, if applicable.	Methods (Key Definitions & Comorbidity Assessment)	RECORD 7.1: A complete list of codes and algorithms used to classify exposures, outcomes, confounders, and effect modifiers should be provided. If these cannot be reported, an explanation should be provided.	Methods (Patient Identification, Key Definitions, Comorbidity Assessment); Supplementary Tables 1, 4, 5, 8, and 11

Data sources/ measurement	8	For each variable of interest, give sources of data and details of methods of assessment (measurement). Describe comparability of assessment methods if there is more than one group	Methods (Data Source & Key Definitions)		
Bias	9	Describe any efforts to address potential sources of bias	Methods (Data Preprocessing and Cohort Construction, Comorbidity Assessment, Survival analysis)		
Study size	10	Explain how the study size was arrived at	Methods (Cohort Construction)		
Quantitative variables	11	Explain how quantitative variables were handled in the analyses. If applicable, describe which groupings were chosen, and why	Methods (Data Preprocessing & Comorbidity Assessment)		
Statistical methods	12	(a) Describe all statistical methods, including those used to control for confounding<br>(b) Describe any methods used to examine subgroups and interactions<br>(c) Explain how missing data were addressed<br>(d) *Cohort study* - If applicable, explain how loss to follow-up was addressed<br>(e) Describe any sensitivity analyses	Methods (Data Preprocessing and Cohort Construction & Statistical Analysis)<br><br><br>N/A<br><br>N/A		
Data access and cleaning methods		..		RECORD 12.1: Authors should describe the extent to which the investigators had access to the database population used to create the study population.	Methods (Data Source); Declarations (Availability of data and materials)

				RECORD 12.2: Authors should provide information on the data cleaning methods used in the study.	Methods (Data Source - harmonization of 85 sites into OMOP CDM)
Linkage		..		RECORD 12.3: State whether the study included person-level, institutional-level, or other data linkage across two or more databases. The methods of linkage and methods of linkage quality evaluation should be provided.	N/A (single centralized database, no linkage)
**Results**					
Participants	13	(a) Report the numbers of individuals at each stage of the study (*e.g.*, numbers potentially eligible, examined for eligibility, confirmed eligible, included in the study, completing follow-up, and analysed)<br>(b) Give reasons for non-participation at each stage.<br>(c) Consider use of a flow diagram	Results (first paragraph); Figure 1 & Supplementary Figure 1	RECORD 13.1: Describe in detail the selection of the persons included in the study (*i.e.,* study population selection) including filtering based on data quality, data availability and linkage. The selection of included persons can be described in the text and/or by means of the study flow diagram.	Methods (Cohort Construction)<br>Results (first paragraph); Figure 1 & Supplementary Figure 1
Descriptive data	14	(a) Give characteristics of study participants (*e.g.*, demographic, clinical, social) and information on exposures and potential confounders<br>(b) Indicate the number of participants with missing data for each variable of interest<br>(c) *Cohort study* - summarise follow-up time (*e.g.*, average and total amount)	Table 1; Supplementary Tables 6 and 7<br><br>Results (Matched cohort characteristics); Supplementary Figure 1<br>Table 1		
Outcome data	15	*Cohort study* - Report numbers of outcome events or summary measures over time	Table 1 (Severity Type); Supplementary Table 12		

Main results	16	(a) Give unadjusted estimates and, if applicable, confounder-adjusted estimates and their precision (e.g., 95% confidence interval). Make clear which confounders were adjusted for and why they were included<br>(b) Report category boundaries when continuous variables were categorized<br>(c) If relevant, consider translating estimates of relative risk into absolute risk for a meaningful time period	Table 2; Results (throughout)<br>N/A<br>N/A		
Other analyses	17	Report other analyses done—e.g., analyses of subgroups and interactions, and sensitivity analyses	Results (Comorbidity stratification, survival, acute-phase interventions); Figures 2-4; Supplementary Figures 5-8		
**Discussion**					
Key results	18	Summarise key results with reference to study objectives	Conclusions		
Limitations	19	Discuss limitations of the study, taking into account sources of potential bias or imprecision. Discuss both direction and magnitude of any potential bias	Discussion (paras. 7-9)	RECORD 19.1: Discuss the implications of using data that were not created or collected to answer the specific research question(s). Include discussion of misclassification bias, unmeasured confounding, missing data, and changing eligibility over time, as they pertain to the study being reported.	Discussion (paras. 7-9)
Interpretation	20	Give a cautious overall interpretation of results considering objectives, limitations, multiplicity of analyses, results from similar studies, and other relevant evidence	Discussion (throughout)		

Generalisability	21	Discuss the generalisability (external validity) of the study results	Discussion (para. 10)		
**Other Information**					
Funding	22	Give the source of funding and the role of the funders for the present study and, if applicable, for the original study on which the present article is based	Declarations (Funding)		
Accessibility of protocol, raw data, and programming code		..		RECORD 22.1: Authors should provide information on how to access any supplemental information such as the study protocol, raw data, or programming code.	Declarations (Availability of data and materials)

*Reference: Benchimol EI, Smeeth L, Guttmann A, Harron K, Moher D, Petersen I, Sørensen HT, von Elm E, Langan SM, the RECORD Working Committee. The REporting of studies Conducted using Observational Routinely-collected health Data (RECORD) Statement. *PLoS Medicine* 2015; in press.

*Checklist is protected under Creative Commons Attribution (CC BY) license.
